# An efficient algorithm for finding all possible input nodes for controlling complex networks

**DOI:** 10.1038/s41598-017-10744-w

**Published:** 2017-09-06

**Authors:** Xizhe Zhang, Jianfei Han, Weixiong Zhang

**Affiliations:** 1 Key Laboratory of Medical Image Computing of Northeastern University, Ministry of Education, Shenyang, China; 2 School of Computer Science and Engineering, Northeastern University, Shenyang, Liaoning China; 3 College of Math and Computer Science, Institute for Systems Biology, Jianghan University, Wuhan, 430056 China; 4 Department of Computer Science and Engineering, Washington University, Saint Louis, Missouri USA

## Abstract

Understanding structural controllability of a complex network requires to identify a Minimum Input nodes Set (*MIS*) of the network. Finding an *MIS* is known to be equivalent to computing a maximum matching of the network, where the unmatched nodes constitute an *MIS*. However, maximum matching is often not unique for a network, and finding all possible input nodes, the union of all *MIS*s, may provide deep insights to the controllability of the network. Here we present an efficient enumerative algorithm for the problem. The main idea is to modify a maximum matching algorithm to make it efficient for finding all possible input nodes by computing only one *MIS*. The algorithm can also output a set of substituting nodes for each input node in the *MIS*, so that any node in the set can replace the latter. We rigorously proved the correctness of the new algorithm and evaluated its performance on synthetic and large real networks. The experimental results showed that the new algorithm ran several orders of magnitude faster than an existing method on large real networks.

## Introduction

Controlling complex networks^1–3^ is of great importance in many applications, such as social, biological and technical networks. For example, it has been shown that understanding network controllability can help identify genes responding to viral infection^4^ and genes related to cancer^5^, as well as assist analyzing metabolic process^6^.

A network is said to be controllable if it can be driven from any initial state to a desirable state in finite steps by exerting external control signals on some selected nodes^1^, which are called driver nodes^7^, input nodes^8^ or control nodes^9^. The controllability of a network can be determined by Kalman’s controllability rank condition^1^ if the weight of each edge is known, or Lin’s structural controllability theory^10^ if only a zero or non-zero value of each edge’s weight is known. As edge weights of many real networks cannot be precisely measured and are often unknown, the structural controllability theory has been widely adopted recently to analyze biological systems, such as protein interaction network^4, 5, 11^, signaling pathway network^12^, and gene regulatory network^13, 14^.

Based on the structural controllability theory, one approach^7–9, 15, 16^ assumes that a node can control only one of its outgoing neighbor. Therefore, input nodes of a network can be inferred by finding a maximum matching of the network, which is consisted of the set of maximum edges that do not share nodes^3^. The unmatched nodes related to a maximum matching constitute a Minimum Input nodes Set, or *MIS*. However, this approach can be only applied to directed networks. Another approach^5, 17, 18^ assumes that a node can control all of its outgoing edges independently, that is, a node can output different signals for each edge. Under this assumption, a minimum node dominate set (*MDS*) can be used to control the network^18^. This approach may be more reasonable for artificial networks and can be applied to undirected networks.

Based on above frameworks, extensive works have been done about control principles of complex networks. It has been shown that the size of an *MIS* is closely related to the node degree distribution of the network^7^. Interestingly, the fraction of input nodes is primarily determined by the nodes of low in- and out-degrees^16^. The concepts of input nodes and *MIS* are also extensively used in analyzing many biological networks, e.g., identifying important proteins in biological networks^4, 5^, analyzing interbank networks^19^, and increasing the effectiveness of selective modulation of brain networks^20^.

**Figure 1:**
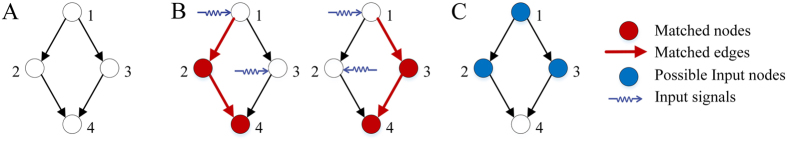
An example network with two *MISs*. (**A**) An example network; (**B**) two *MIS* of the network *D*_1_ = {1, 3}, *D*_2_ = {1, 2}; (**C**) all possible input nodes of the network, which form the union of both *MIS*s.

Unfortunately, maximum matching is not unique for most networks^21^ (Fig. 1), so there may exist numerous *MIS*s. Although these *MIS*s have the same size, they may have different input nodes. We call a node in an *MIS* a *possible input node*. Apparently, all possible input nodes are the union of all *MIS*s. It is important to find all possible input nodes for understanding the controllability of a complex network. For example, finding all possible input nodes may help understand the roles of nodes in control systems^22^, design suitable MISs under different constraints^8^, and identify critical genes on signaling pathways^14^. Finding all possible input nodes is essentially an enumeration problem. While enumeration problems have been extensively studied, such as for enumerating all maximum matchings^23, 24^ or all maximally-matchable edges^25^, there are very few works on how to find all unmatched nodes. To solve this problem, a previous approach^22^ first computes a maximum matching and then assess if an unmatched node is a possible input node by removing it to test if its removal may result in a larger maximum matching. The computational complexity of finding a maximum matching is O(*N* ^1/2^ *L*) and the evaluation process is O(*NL*) on a network of *N* nodes and *L* edges, for a total complexity of O(*NL*) for this method.

We developed an efficient algorithm for finding all possible input nodes of a network. We proved that all possible input nodes could be identified by a simple modification to a maximum matching algorithm. The complexity of our algorithm is *O*(*N* ^1/2^ *L*), which is the same as the complexity of the maximum matching algorithm. Because our algorithm does not need to evaluate every node of the network, it runs several orders of magnitude faster than the previous method^22^ on large networks. Furthermore, our algorithm can also output the substituting nodes set for each input nodes. Because some input nodes may not be suitable to be inputted control signals due to some economic or technical constraints, these substituting nodes can be used to replace the original input nodes and obtain new *MISs* with some input nodes replaced.

## Method

**Figure 2:**
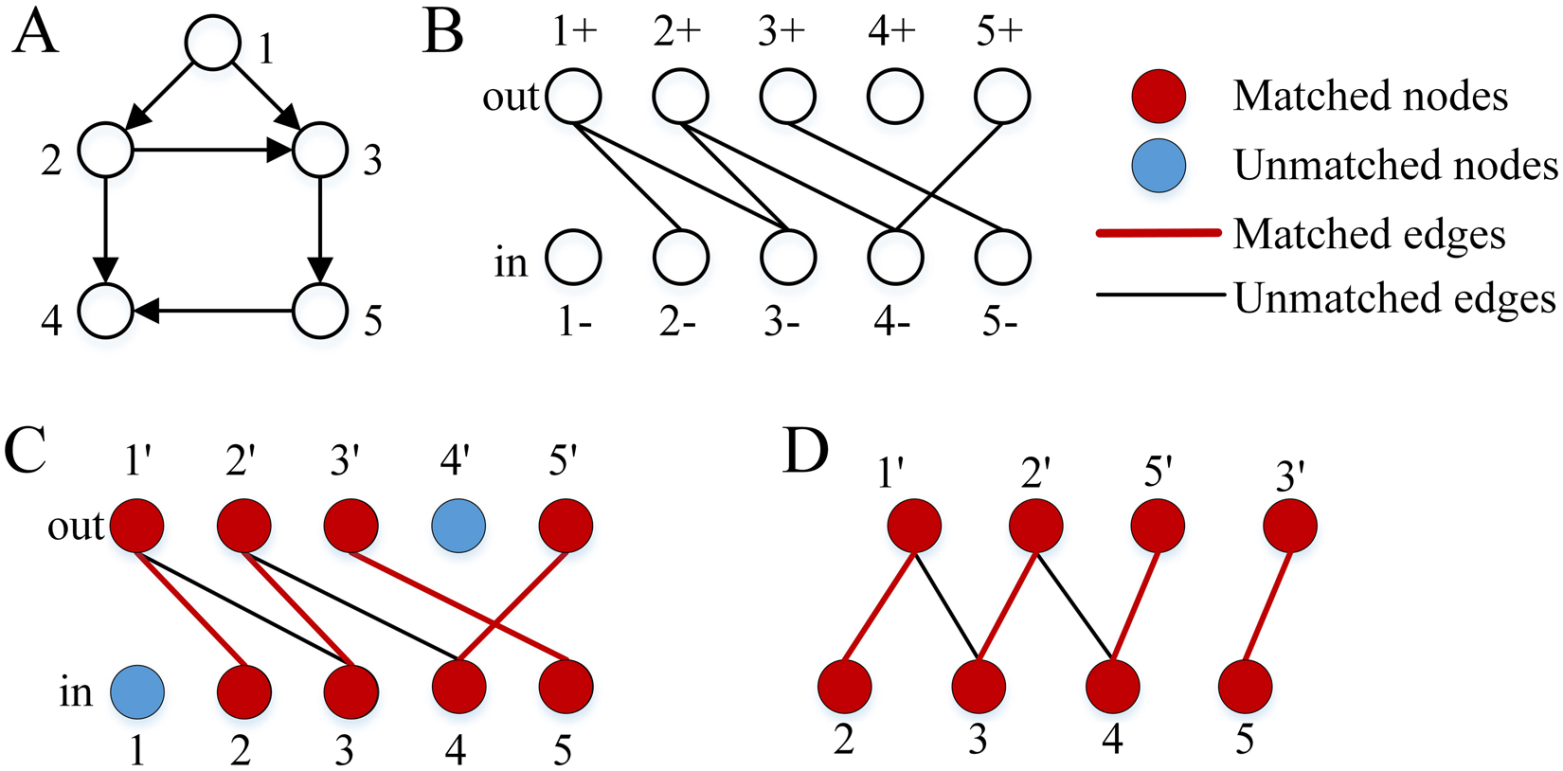
An example of a maximum matching of a network. (**A**) A directed network; (**B**) its corresponding bipartite graph; (**C**) A maximum matching of the bipartite graph. An unmatched node in the in-set is an input node; (**D**) Two alternating paths corresponding to the maximum matching in (**C**).

Consider a directed network *G*(*V, E*) over a set of nodes *V* and a set of edges *E*. To find an *MIS* of a directed network *G*(*V, E*), we first convert *G*(*V, E*) to an equivalent undirected bipartite graph *B*(*V* ^*in*^ *, V* ^*out*^ *, E*) (Fig. 2A,B). The bipartite graph is built by splitting the node set *V* into two node sets *V* ^*in*^ and *V* ^*out*^, where a node *n* in *G* is converted to two nodes *n* ^*in*^ and *n* ^*out*^ in *B*, and nodes *n* ^*in*^ and *n* ^*out*^ are, respectively, connected to the in-edges and out-edges of node *n*.

Now consider maximum matching. A *matching* is a set of edges that share no common node. A node is called a *matched node* if it is connected to a matching edge, or *unmatched node*, otherwise. A matching with the maximum number of edges is called a *maximum matching*. In an undirected bipartite graph, an *alternating path* is a path whose edges are alternate in and out matching. An *augmenting path* is an alternating path whose two end nodes are unmatched nodes. Based on the Berge theorem^26^, a matching *M* ^*^ is a maximum matching if there is no augmenting path in *B*(*v*_1_ *, v*_2_ *, E*) with respect to *M* ^*^. The input nodes are the unmatched nodes in *V* ^*in*^ corresponding to a maximum matching of bipartite graph *B*(*V* ^*in*^, *V* ^*out*^, *E*). The unmatched nodes in *V* ^*in*^ corresponding to any maximum matching form an MIS of *G*. If there exists a perfect matching in the network, the *MIS* can be any node of the network based on the Minimum Input Theorem^7^.

Because maximum matching is not unique for most networks, there may exist many *MISs*. The union of all *MISs* contains all possible input nodes. We now show that to find all possible input nodes of a network, we only need to compute one maximum matching without enumerating all *MISs* or evaluating all matched nodes as done before in ref. 22.

**Theorem 1:** Given a directed network *G* and a maximum matching *M* of *G*, a node *n* is a possible input node if it satisfies one of the following two conditions:

1. Node *n* is an input node related to *M*;
2. The in-node *n* ^*in*^ of the bipartite graph *B* can be reached from an input node *m* ^*in*^ related to *M* through an alternating path *p*_*nm*_.

**Proof:** It is sufficient to prove condition 2.

Sufficiency. Suppose that node *n* satisfies condition 2. Apparently, the length of *p*_*nm*_ must be even because both node *n* ^*in*^ and *m* ^*in*^ are in the set *V* ^*in*^ of bipartite graph *B*. Therefore, the alternating path *p*_*nm*_ must start with an unmatched edge connected to *m* ^*in*^ and end with a matched edge connected to node *n* ^*in*^. Change the types of all edges of *p*_*nm*_, i.e., change the matched edges to unmatched and the unmatched edges to matched, then the new path *p*’_*nm*_ is still an alternating path. Consequently, we get a new maximum matching *M*’. Clearly, the node *n* ^*in*^ is not matched by *M*’ and *m* ^*in*^ is matched by *M*’. Therefore, node *n* is an input node of *MIS D*’ = *D*-{*m*} + {*n*}.

Necessity. Let node *n* ^*in*^ be matched in *M* and be unreachable by any input node related to *M*. Suppose that node *n* ^*in*^ is not matched by a maximum matching *M*’. Node *n* ^*in*^ must have at least one in-edge because it is matched by *M*. Therefore, there must be an alternating path *p*_*nm*_ related to *M*’ which starts with unmatched node *n* ^*in*^ and end with a matched node *m* ^*in*^. Now consider the path *p*_*nm*_ under the maximum matching *M*. The length of *p*_*nm*_ must be even because nodes *n* ^*in*^ and *m* ^*in*^ are both in the set *V* ^*in*^. Therefore, the alternating path *p*_*nm*_ must end with an unmatched node *m* ^*in*^ related to *M* because *n* ^*in*^ is matched by *M*. This contradicts the fact that *n* ^*in*^ cannot be reached by any input node related to *M*, which completes the proof.

The above proof implies an important fact that any of the nodes reachable from an input node can be used to replace the original input node and obtain a new *MIS*. Therefore, we have the following corollary:

**Corollary 1:** Consider an *MIS D* and one of its input node *m* ∈ D, let *K*_*m*_ be the set of the nodes that satisfied condition 2 of Theorem 1. For every node *n* ∈ *K*_*m*_, *D*’ = *D* − {*m*} + {*n*} is an *MIS*.

**Figure 3:**
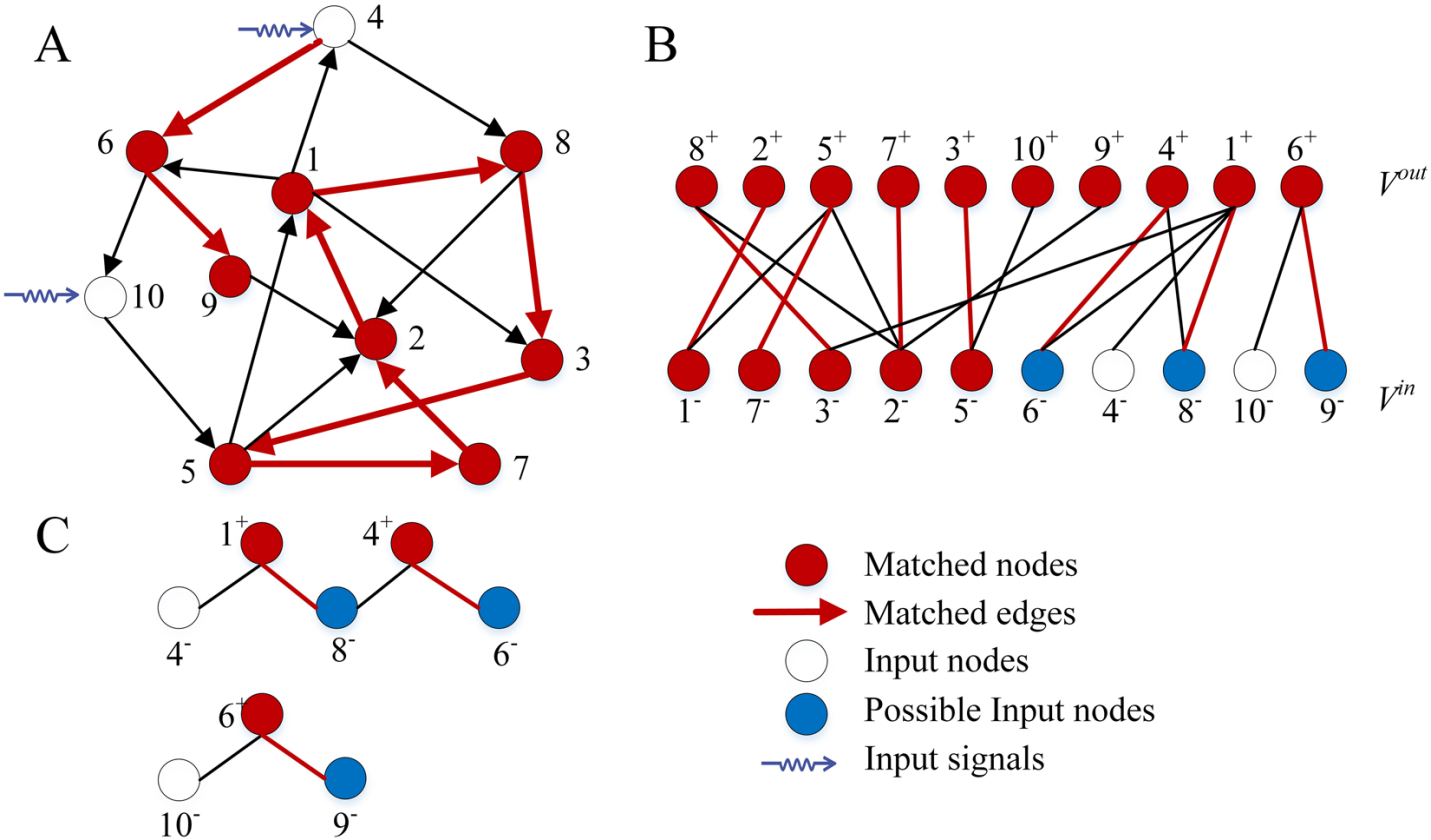
An example of the process of algorithm *All_Input*(*G*). (**A**) A sample network and its maximum matching (red edges) (**B**) the corresponding bipartite graph; (**C**) In the last step of the algorithm, we search for alternating paths from unmatched nodes. The nodes on the alternating paths in *V* ^*in*^ are nodes {8, 6, 9}, and the input nodes are {4, 10}. Therefore, all possible input nodes of the network are {4, 6, 8, 9, 10}. The substituted nodes of input node 4 are node 8 and node 6, and those of input node 10 is node 9. Therefore, if node 4 or 10 is not suitable to be inputted control signals, we can use the corresponding substituted nodes to input control signals and regain full control of the network.

Based on Theorem 1, the proof to the corollary is trivial. Corollary 1 suggests how to find the substituting nodes for an input node. For example, in Fig. 3, the substituting nodes of input node 4 are node 8 and node 6. If node 4 is not suitable for accepting control signals, we can use either node 8 or node 6 to substitute node 4, and the new *MIS* would be {8, 10} or {6, 10}.

The significance of Theorem 1 and Corollary 1 is that all possible input nodes can be identified by some alternating paths of the input nodes of any given *MIS*. This observation leads to a novel two-step approach to identification of all possible input nodes, i.e., we first compute an *MIS* and then consider all of its alternating paths. Moreover, these two steps can be combined using a simple modification to the Hopcroft–Karp maximum matching algorithm for undirected graphs^27^. The basic idea of the Hopcroft–Karp algorithm is to iteratively find all augmenting paths corresponding to the matching *M* at hand, and then to derive a larger matching *M*’. A maximum matching is obtained when no augmenting path can be founded. The last step of the algorithm is exactly to look for all alternating paths starting from the input nodes of the maximum matching. Therefore, all possible input nodes can be obtained in the last step of Hopcroft–Karp algorithm based on Theorem 1.

The above idea and steps can be formulated in Algorithm *All_Input*(*G*) for finding all possible input nodes in network *G*, which is listed as follows:

All_Input(G):

1. For a directed network *G*(*V*, *E*), let *B*(*V* ^*out*^, *V* ^*in*^, *E*) be its corresponding bipartite graph; let the initial matching *M* = null;
2. Find all the alternating paths of all unmatched nodes in *V* ^*in*^, denoted as *AP* = {*P*_1_, *P*_2_ … *P*_*n*_}, and let the nodes of *AP* in *V* ^*in*^ as candidate results;
3. If *AP* contain augmenting paths, expand all augmenting paths and obtain a new matching *M*’; clear all candidate nodes, *M* = *M*’; return to step 2;
4. If *AP* contain no augmenting path, the candidate nodes are all possible input nodes, and the set of the unmatched nodes is an *MIS* of *G*.

Figure 3 illustrates an example of *All_Input*(*G*) on a small network. The time complexity of the above algorithm is the same as that of the Hopcroft-Karp algorithm, which is *O*(*N* ^1/2^ *L*).

## Result

To assess the efficiency of the new algorithm, which was coded in JAVA, we compared it with the previous algorithm^22^. The source code of our algorithm and the previous algorithm is available in the supplementary information. The comparison was done on a Windows 7 workstation with a quad-core Intel i7-3770 processor of 3.9 GHz and 32GB DDR3 1600MHz memory.

**Figure 4:**
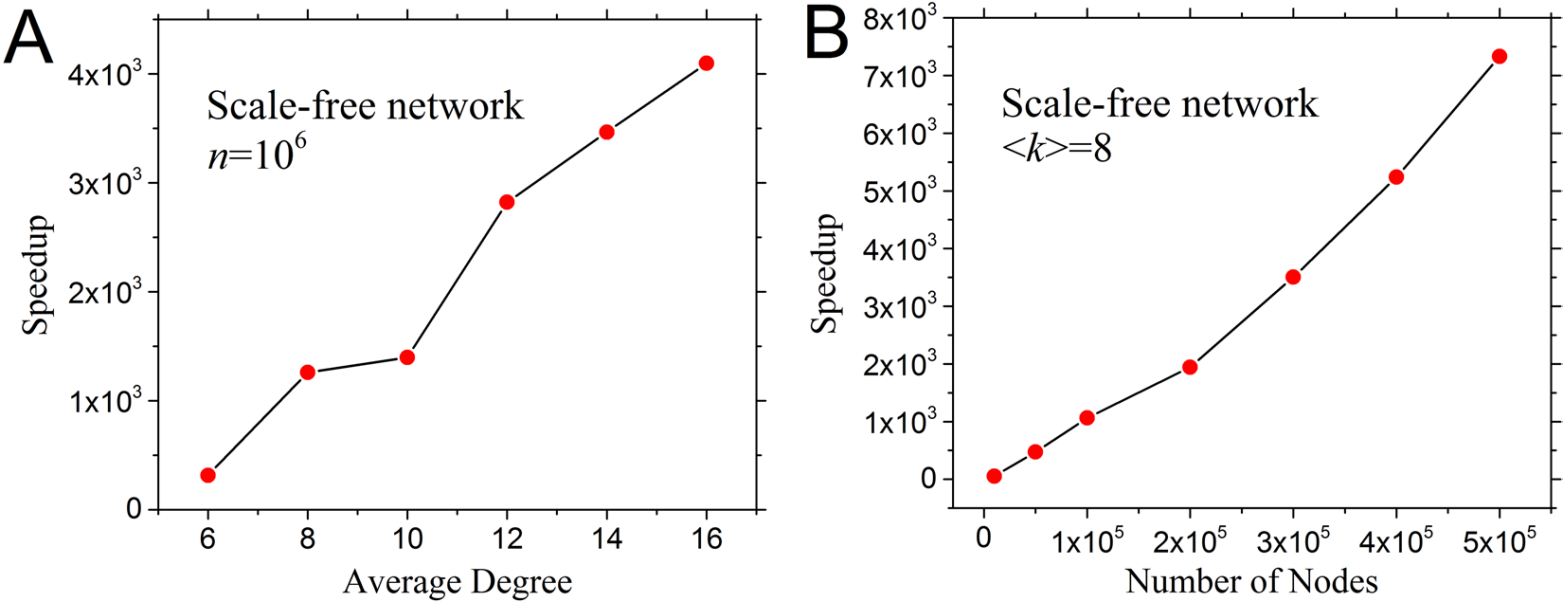
Speedup of our algorithm as compared to the previous algorithm^22^. (**A**) Speedup as a function of the average degree when *n* = 10^6^; (**B**) Speedup as a function of the number of nodes when average degree < *k* > = 8. The networks are generated based on Scale-Free Network model^29^ with *r* ^*in*^ = *r* ^*out*^ = 3.

We considered 13 synthetic networks, in which the number of nodes *n* varied from 10^5^ to 5 × 10^6^ and the average degree <*k*> varied from 6 to 16. Networks were generated with Gephi^28^ based on the Scale-Free Network model^29^. The experimental results on these networks showed that our algorithm outputted the same results set yet significantly outperformed previous algorithm^22^ (Fig. 4). With a small network with *n* = 10^5^, our algorithm achieved 52x speedup compared to the previous algorithm^22^. With a larger network with *n* = 5 × 10^6^, our algorithm achieved 7330x speedup with the execution time being only 3.276 seconds. Note that the speedup increases with the average degree.<*k*> (Fig. 4A), which indicates that our algorithm has better performance on dense networks. The details of the results are listed in Table 1.

**Table 1:** Comparison of the execution time of some synthetic networks.

*Network*		*L*	*n*_*pd*_	*t*_*new*_ *(sec)*	*t*_*old*_ *(sec)*	*Speedup*
*n* = 10^6^	<*k*> = 6	300000	0.444	0.343	108.1	315.1
	<*k*> = 8	400000	0.396	0.546	687.9	1260.1
	<*k*> = 10	500000	0.124	0.858	1198.6	1396.9
	<*k*> = 12	600000	0.039	0.826	2330.6	2821.5
	<*k*> = 14	700000	0.018	0.889	3080.3	3464.9
	<*k*> = 16	800000	0.008	0.952	3900.1	4096.8
<*k*> = 8	*n* = 10^5^	40000	0.332	0.047	2.5	52.1
	*n* = 5 * 10^5^	200000	0.388	0.218	102.9	472.0
	*n* = 10^6^	400000	0.411	0.530	562.9	1062.2
	*n* = 2 * 10^6^	800000	0.397	1.435	2784.6	1940.5
	*n* = 3 * 10^6^	1200000	0.397	2.106	7381.4	3504.9
	*n* = 4 * 10^6^	1600000	0.399	2.777	14550.0	5239.5
	*n* = 5 * 10^6^	2000000	0.395	3.276	24012.7	7329.9

For each network, we show its average degree <*k*>, number of nodes (*N*) and edges (*L*), destiny of all possible input nodes *N*_*pd*_, the execution time of our method *t*_*new*_, the execution time of previous algorithm^22^ *t*_*old*_, and the speedup ratio.

Next, we evaluated the performance of the algorithm on some real networks. These networks were selected based on their diversity of topological structures. These networks include biological networks, social networks, and Internet networks. The size of these networks varied from very small (*E. Coli* network, 423 nodes) to very large (Amazon network, 4 × 10^6^ nodes). The results shown in Table 2 indicated that our algorithm also significantly outperformed the previous algorithm. On large networks, such as Amazon or Twitter, the results can be obtained within 10 seconds, resulting in an almost 10^4^x speedup compared to the previous algorithm.

**Table 2:** Comparison of the execution time of some real networks.

Type	Name	*N*	*L*	*n*_*pd*_	*t*_new_ (*sec*)	*t*_*old*_ (*sec*)	*Speedup*
Biological	*E. Coli* ^30^	423	578	0.730	0.001	0.016	16.0
	TRN-Yeast-1^31^	4441	12873	0.999	0.015	0.062	4.1
	TRN-Yeast-2^32^	688	1079	0.920	0.001	0.015	15.0
	Human PPI^33^	6339	34814	0.585	0.032	1.295	40.5
Trust	Slashdot0902^34^	82168	948464	0.912	0.421	1568.3	3725.2
	Slashdot0811^34^	77360	905468	0.910	0.234	1388.9	5935.5
	WikiVote^35^	7115	103689	0.666	0.047	2.044	43.5
	SciMet^36^	3084	10416	0.661	0.015	0.187	12.5
	Kohonen^37^	4470	12731	0.669	0.016	0.172	10.8
Internet	p2p-1^38^	10876	39994	0.911	0.062	2.746	44.3
	p2p-2^38^	8846	31839	0.926	0.046	1.732	37.6
	p2p-3^38^	8717	31525	0.933	0.031	1.700	54.8
Product co-purchasing	Amazon0302^39^	262111	1234877	0.177	1.685	14119.4	8379.5
	Amazon0312^39^	400727	3200440	0.127	9.344	36696	3927.2
	Amazon0505^39^	410236	3356824	0.915	7.519	45453	6045.2
	Amazon0601^39^	403394	3387388	0.053	2.886	49293	17080.1
Social network	Twitter^40^	81306	1768149	0.800	2.230	2532.5	1135.6
	Higgs_Twitter^41^	456626	14855842	0.297	12.589	66445.2	5278.0
	UClonline^42^	1899	20296	0.819	0.016	0.296	18.5
	Facebook_348^40^	572	6384	0.612	0.001	0.062	62.0

For each network, we show its type, name, number of nodes (*N*) and edges (*L*), density of all possible input nodes *n*_*pd*_, the execution time of our method *t*_*new*_, the execution time of previous algorithm^22^ *t*_*old*_, and the speedup ratio.

**Figure 5:**
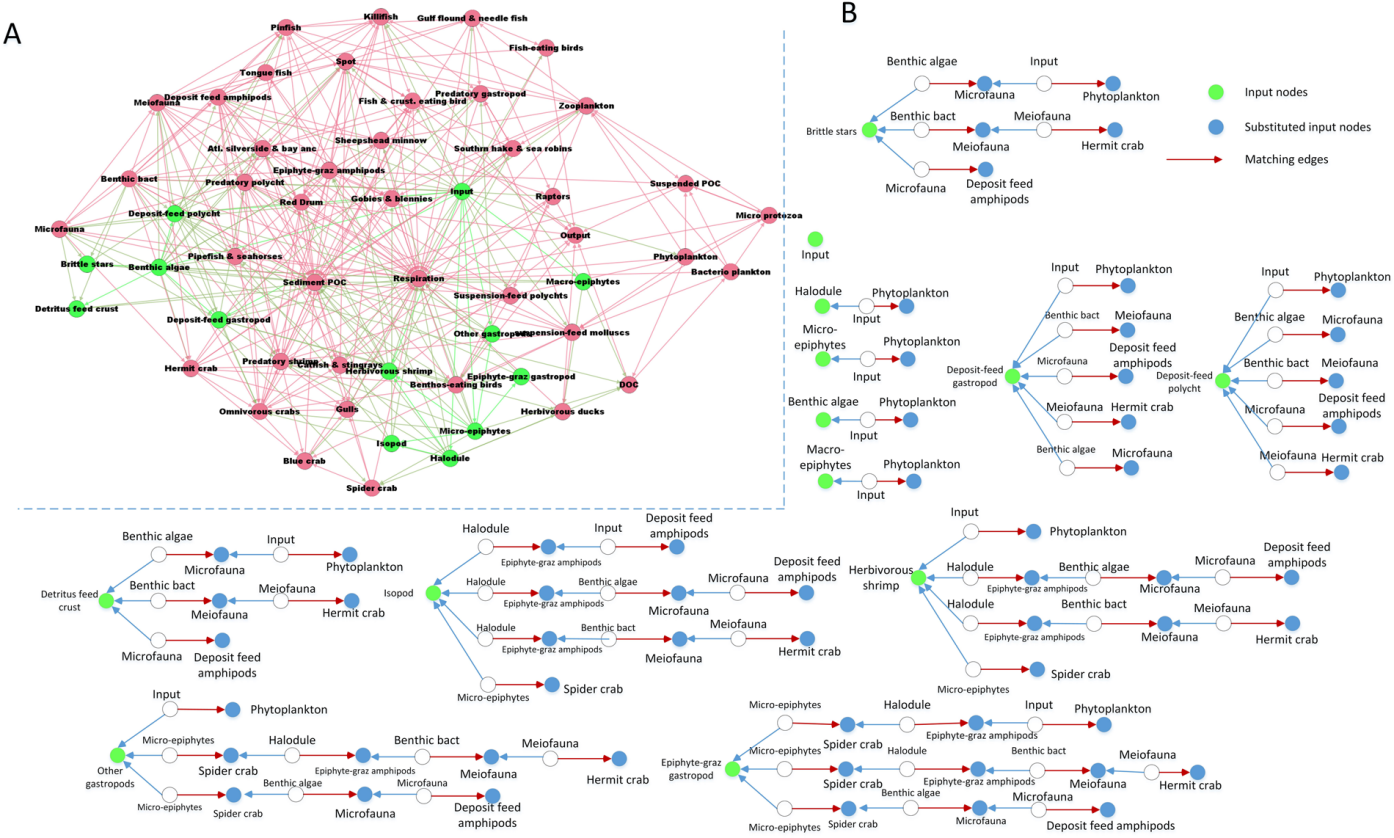
Input nodes and their substituted set of St. Marks foodweb^43^. (**A**) Network topology of St. Marks foodweb. The input nodes are green nodes. (**B**) All 13 Input nodes and their substituted nodes. We showed the alternating paths which connected the input nodes and their substituted nodes. The red edges are the matching edges.

**Figure 6:**
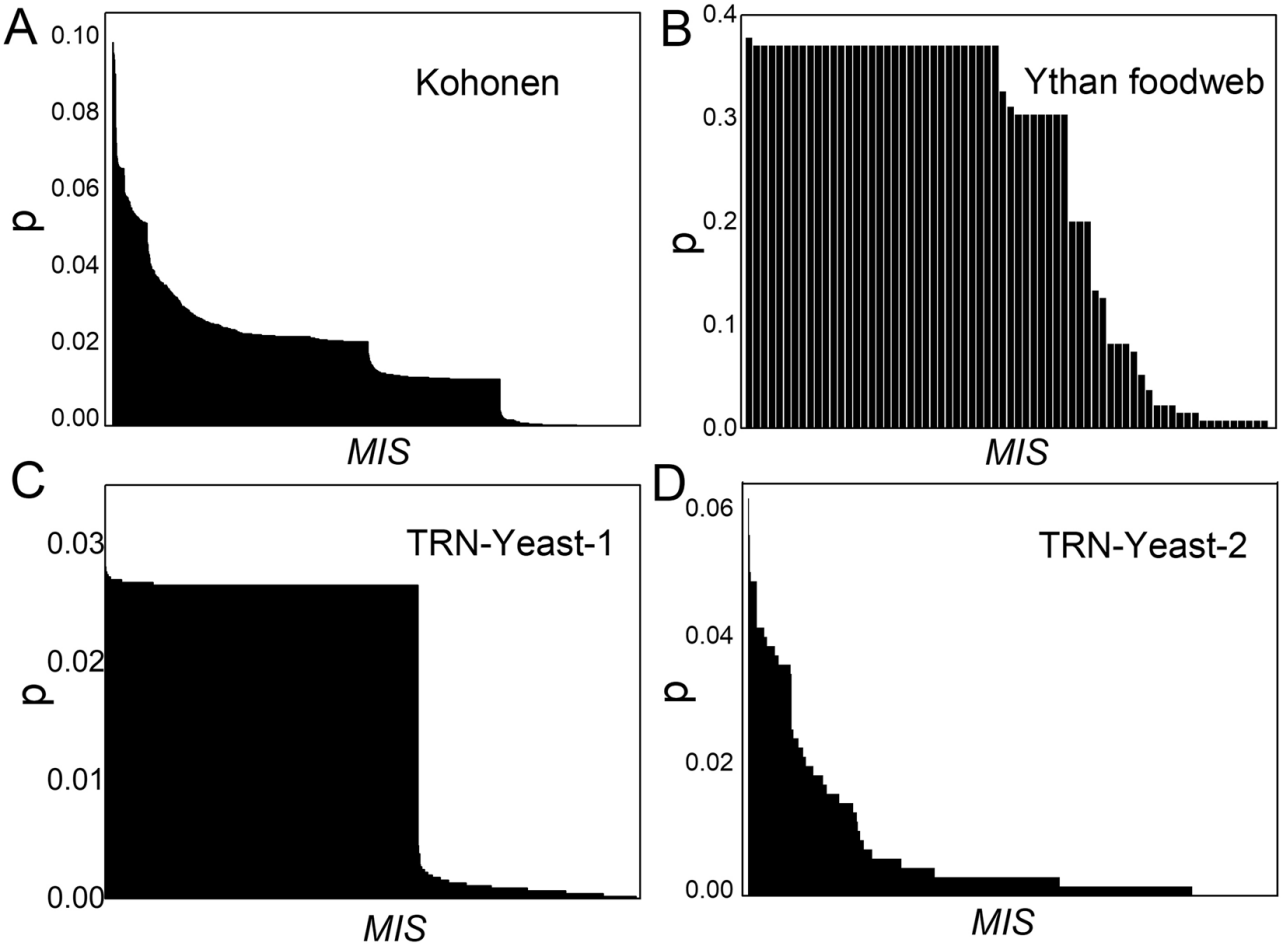
Percentage of substituted nodes for each input node of real networks. The networks we used are Kohonen^37^, TRN-Yeast-1^31^, Ythan foodweb^44^ and TRN-Yeast-2^31^. The vertical axis represents the percentage of substituted nodes *p*_*i*_ = *s*_*i*_/*N*, where *s*_*i*_ is the number of substituted nodes of input node *i*, and *N* is the total number of the nodes. The horizontal axis shows an *MIS*, in which the input nodes are sorted based on *s*_*i*_ by descending order.

As we have proven in corollary 1, our algorithm can also output the substituting nodes for each input node. Figure 5A shows an example of St. Marks foodweb^43^, which has 13 input nodes in an *MIS*. We computed the substituting nodes for each input node and showed the alternating paths between them in Fig. 5B. Interestingly, the size of the substituting nodes set of each input node is different, indicating some input nodes are more robust in controlling the network. The experimental results on other real networks are similar (Fig. 6). Note that some input nodes have the same number of substituting nodes because they are linked to the same set of substituting nodes, e.g., those of TRN-Yeast-1^31^ and Ythan foodweb^44^ in Fig. 6.

## Conclusion

We developed an efficient algorithm for finding all possible input nodes for controlling complex networks. We proved that all possible input nodes can be efficiently identified along with computing an *MIS* without increasing the overall complexity beyond finding the *MIS*. Therefore, our algorithm offers a significant speedup over the previous algorithm on both synthetic networks and many large real networks. Furthermore, our algorithm can also output the substituted nodes set for each input node. It means that once we computed an *MIS*, we can immediate obtain all the substituting nodes for the *MIS*. Thanks to its efficiency, the new algorithm makes it possible to study controllability of large real-world networks and will have many potential applications in diverse areas.

### Data availability

All data generated or analyzed during this study are included in this article (and its Supplementary Information files).

## Electronic supplementary material

Source code 1

Source code 2
